# Domain Structure, Thermal and Mechanical Properties of Polycaprolactone-Based Multiblock Polyurethane-Ureas under Control of Hard and Soft Segment Lengths

**DOI:** 10.3390/polym14194145

**Published:** 2022-10-03

**Authors:** Alexander N. Bugrov, Yulia E. Gorshkova, Elena M. Ivan’kova, Gennady P. Kopitsa, Alina A. Pavlova, Elena N. Popova, Valentina E. Smirnova, Ruslan Y. Smyslov, Valentin M. Svetlichnyi, Gleb V. Vaganov, Boris V. Vasil’ev

**Affiliations:** 1 Institute of Macromolecular Compounds of Russian Academy of Sciences, V.O., Bol’shoy pr. 31, 199004 St. Petersburg, Russia; 2 Department of Physical Chemistry, Saint Petersburg Electrotechnical University (ETU “LETI”), Str. Professora Popova 5, 197376 St. Petersburg, Russia; 3 Ioffe Institute, Str. Politechnicheskaya 26, 194021 St. Petersburg, Russia; 4 Joint Institute for Nuclear Research, Joliot-Curie 6, 141980 Dubna, Russia; 5 Petersburg Nuclear Physics Institute NRC KI, 188300 Gatchina, Russia

**Keywords:** microphase separation, thermoplastic elastomers, relaxation and phase transitions, shape memory effect, supramolecular structure, domain temperature evolution

## Abstract

A series of multiblock polyurethane-ureas (PUU) based on polycaprolactone diol (PCL) with a molecular mass of 530 or 2000 g/mol, as well as hard segments of different lengths and structures, were synthesized by the step-growth polymerization method. The chemical structure of the synthesized multiblock copolymers was confirmed by IR- and NMR-spectroscopy. Differential scanning calorimetry (DSC) and dynamic mechanical analysis (DMA) were used to determine the relaxation and phase transition temperatures for the entire series of the obtained PUU. The X-ray diffraction (XRD) method made it possible to identify PUU compositions in which the crystallizability of soft segments (SS) is manifested due to their sufficient length for self-organization and structuring. Visualization of the crystal structure and disordering of the stacking of SS with an increase in their molecular mobility during heating are shown using optical microscopy. The change in the size of the hard phase domains and the value of the interdomain distance depending on the PCL molecular mass, as well as the length and structure of the hard block in the synthesized PUU, were analyzed using small-angle X-ray scattering (SAXS) and small-angle neutron scattering (SANS). The evolution of the domain structure upon passing through the melting and crystallization temperatures of PUU soft blocks was studied using SANS. The studies carried out made it possible to reveal the main correlations between the chemical structure of the synthesized PUU and their supramolecular organization as well as thermal and mechanical properties.

## 1. Introduction

For the first time, linear multiblock polyurethanes (PU) were synthesized by Schollenberger [1,2] in 1958 and became widespread due to the possibility of controlling their deformation and strength properties from rubber-like materials to plastics [3,4]. Multiblock PU macromolecules, as a rule, consist of alternating soft and hard segments chemically bonded to each other along the main chain [5]. Hard segments are formed by diisocyanates and short-chain diols and/or diamines, the glass transition temperature (T_g_) of which is quite high. SS are represented by long-chain diols, and their T_g_ is much lower than room temperature. The different nature of thermodynamically incompatible blocks leads to their microsegregation and the formation of a domain structure [6,7,8,9,10]. Ordered areas of block structure (domains) in PU can be formed both due to the stacking of hard segments through intermolecular interactions, and as a result of mutual orientation of soft polyesters, if they are of sufficient length. SS provide elasticity to multiblock PU, and their structured regions are used to switch between phases. The hard blocks act as physical crosslinks and allow the multiblock copolymer to return to its original state. Such an organization of segmented PU often makes it possible to implement the shape memory effect (SME) in them. For its manifestation, as a rule, it is necessary to have the coexistence of a phase fixing the shape of the polymer through chemical or physical crosslinks, as well as a phase allowing the reversibility of thermodynamic states through relaxation or phase transitions, when the system passes from low to high molecular mobility [11,12,13,14].

The degree of microphase separation, the size distribution of the domains, the stacking nature of the hard and soft segments, as well as their ratio, determine the morphological organization of multiblock PU [15,16]. In turn, the structural and morphological features of the interaction between the hard and soft phases affect the thermal and mechanical characteristics of segmented PU [17]. The regulation of the microphase structure of multiblock copolymers is usually carried out by choosing the polymerization method, the chemical composition and molecular mass (M_n_) of the monomers used, as well as the post-synthetic heat treatment conditions [18,19,20].

From the point of view of the ability to control the microdomain structure of multiblock PU, new prospects are opened up for obtaining adaptive functional materials based on them, namely, intelligent medical devices [21], implants for minimally invasive surgery [22], self-tightening suture threads [23], miniature manipulators [24], “breathable clothing” [25], etc. However, understanding the phase behavior of such systems is only the first step on the way to the targeted physicochemical design of multiblock copolymers with preprogrammed properties. Adjusting the molecular composition of segmented PU can affect both the type and size of the domains of corresponding bulk morphologies during self-assembly in the condensed state and determine the set of thermal, deformation-strength, and thermomechanical properties that the synthesized multiblock copolymer will possess [26].

To date, a number of works have considered the influence of the type of SS, their molecular mass, and degree of crystallinity on the mechanical properties of multiblock PU. In [27,28], it is shown that an increase in the M_n_ of soft segments in the absence of any other structural changes leads to a decrease in the elastic modulus (*E*) and T_g_ of multiblock PU. The authors of [29] report an increase in the degree of microphase separation as the length of soft blocks increases. In addition, it is noted that the inclusion of semi-crystalline macrodiols, such as PCL, in the structure of multiblock PU promotes an increase in their elastic modulus and tensile strength (σ_br_) while strain at break (ԑ_br_) decreases [30,31]. The mechanical properties of segmented PU are also sensitive to the chemical structure of the soft blocks. For example, the replacement of polyester (poly(ethylene adipate) glycol) with polyethers (poly(oxytetramethylene) glycol (POTM) and poly(oxypropylene) glycol (POP)) in the PU structure leads to a decrease in σ_br_ and *E* values [3]. In the case of polyethers, POTM gave higher σ_br_, *E,* and hardness than POP due to the regularity of its chain structure. It should also be noted that the side chain of the methyl group in POP prevented the crystallization of the SS. According to the literature data, microphase separation is more pronounced in the compositions of segmented PU based on polyether [32].

As well as in the case of the SS, mechanical properties such as strength, strain at break and stiffness depend on the content and chemical composition of the hard segments [33,34]. The authors of the study [35] noted that an increase in the content of the hard segments leads to an enlargement of the hard domains, and, consequently, an increase in *E* and σ_br_ as well as a more pronounced microphase separation. Hard domains usually act as fillers reinforcing the amorphous matrix of SS and also serve as the pseudo-net nodes or as physical cross-links. The chemical composition and symmetry degree of the hard segments affect the PU mechanical properties [36,37]. When using 4,4′-methylene diphenyl diisocyanate (4,4′-MDI), one observes a lower defreezing temperature of segmental mobility of the soft blocks in PU than that for the same compositions based on toluene 2,4-diisocyanate (2,4-TDI). At the same time, the latter compositions have much lower stiffness and strength, and exhibit behavior typical of elastomers [38].

Phase separation in segmented PU has been investigated using a wide range of methods of physicochemical analysis such as DMA, DSC, broadband dielectric and infrared spectroscopy, SANS and SAXS, optical, atomic force, scanning and transmission electron microscopies, as well as solid-state nuclear magnetic resonance [17,39,40,41,42]. However, all these methods are sensitive to various sample properties, such as changes in electron and proton density, mechanical response, or molecular mobility density. Therefore, it is of great importance to cross-examine samples from different points of view in order to confirm the interpretation of their structural characteristics.

This article is aimed at solving the relevant problem of modern material science related to the development of new multiblock polyurethanes. Targeted synthesis of materials based on segmented block copolymers with pre-programmed thermomechanical behavior requires a thorough investigation of the effect of the length and chemical structure of the hard and soft blocks on microphase separation processes. An important feature of multiblock copolymers is the formation of a domain structure, which largely determines their thermal and deformation-strength properties. The main purpose of the present work was to synthesize a number of new multiblock polyurethane-ureas by the method of stepwise addition reaction, where PCL diol with a molecular mass of 530 or 2000 g·mol^−1^ acts as SS, and the hard blocks surrounding it are formed by combining as 2,4-TDI, as 4,4′-MDI with m-phenylenediamine or (4,4′-bis(4″-aminophenoxy)biphenyl sulfone. Taking the obtained PUU compositions as an example, the influence of the molecular mass of the soft blocks, the structure and length of the hard segments on the morphological organization as well as the thermal and mechanical properties of multiblock copolymers were studied with the help of complementary methods of physicochemical analysis.

## 2. Materials and Methods

### 2.1. Materials

**Figure 1:**
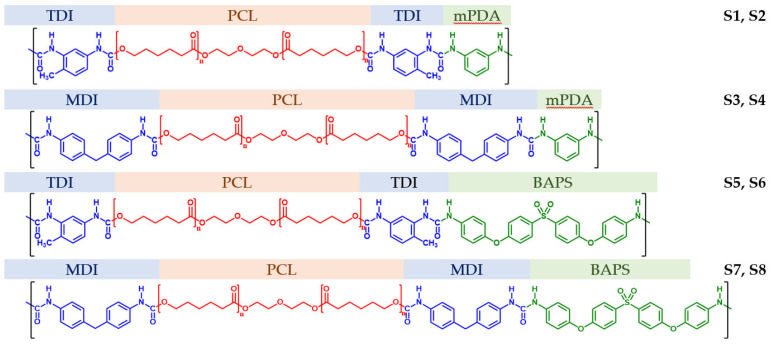
Chemical formulas of segmented PUU synthesized during step-growth polymerization according to the two-step scheme: S1–S8. PCL molecular masses for S1, S3, S5, S7 are 530, and 2000 g·mol^−1^ for S2, S4, S6, S8.

The synthesis of multiblock PUU was carried out by step-growth polymerization in two stages. At the first stage, PCL with a molecular mass of 530 or 2000 g·mol^−1^ (CAS: 36890-68-3, Sigma-Aldrich, St. Louis, MO, USA) acting as an SS was terminated in the melt with diisocyanate containing one (2,4-TDI, CAS: 584-84-9, Sigma-Aldrich) or two (4,4′-MDI, CAS: 101-68-8, 98%, Sigma-Aldrich) aromatic rings. At the second stage, a solution in *N,N*-dimethylformamide (DMFA, CAS: 68-12-2, chemically pure, “Vekton”, Russia) of diamine with one (m-phenylenediamine (mPDA), CAS: 108-45-2, 99%, Sigma-Aldrich) or four aromatic rings (4,4′-bis(4″-aminophenoxy)biphenyl sulfone (BAPS), CAS: 13080-89-2, >98%, TCI, Tokyo, Japan) was added to the resulting macrodiisocyanate melt as a chain extender. All used reagents were preliminarily dried under vacuum at a temperature below the melting point by 20 °C during the day. The amide solvent was preliminarily distilled under vacuum (2–5 mm Hg) before synthesis. Thus, alternately combining aliphatic diols of different molecular mass with diisocyanates and diamines of different chemical structures, we have obtained eight compositions of multiblock PUU; their chemical structure is shown in Figure 1.

### 2.2. Synthesis of Segmented PUU

**Figure 2:**
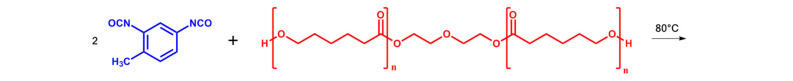

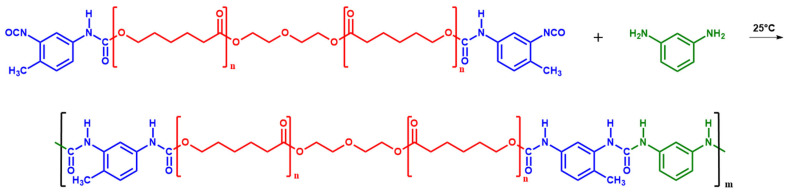
Scheme of reactions for the multiblock PUU synthesis on the example of S1 and S2 compositions.

Diisocyanate and polycaprolactone diol were loaded into a three-necked round-bottom flask equipped with a stirrer and dropping funnel in an argon atmosphere in a ratio of 2:1. The contents of the flask were intensively stirred at a temperature of 80 °C, which allowed a homogeneous reaction mixture to be obtained. In the resulting reaction mixture, a reaction between diisocyanate and diol was carried out for 1 h. As a result, on the basis of PCL, a macromonomer containing urethane bonds with two terminal isocyanate functional groups was formed in the melt (Figure 2). The macrodiisocyanate melt was cooled to room temperature, and a low molecular mass diamine, preliminarily dissolved in 3 mL of DMFA, was added in a ratio of 1:1. The diamine solution used as chain extender was added dropwise over 30 min. After that, another 1 mL of DMF was added to the resulting solution through a dropping funnel in order to remove residual diamine solution from the walls of the dropping funnel. The final solution was vigorously stirred for 4 h. During this time, a step-growth polymerization reaction took place between the isocyanate groups of the macromonomer and the amino groups of the chain extender to form urea bonds (Figure 2). Next, the resulting solution of PUU was filtered through a Schott funnel into a test tube with a gas outlet tube using a vacuum pump. The filtered polymer solution was used to obtain films. The formation of the polymer films was carried out from the 20% polymer solution. The solution was applied to a flat glass plate by pouring, after which it was placed in a thermostat and kept at 100 °C for a day to remove the solvent. The thickness of the obtained film was 60 μm.

### 2.3. Methods for Characterizing Segmented PUU

#### 2.3.1. IR Spectroscopy

The chemical structure of the synthesized PUU was confirmed using a Bruker Vertex 70 IR Fourier spectrometer (Bruker, Karlsruhe, Germany) equipped with the ATR (Attenuated Total Reflection) reflector (Pike Technologies, Madison, WI, USA). Zn-Se crystals in the form of prisms with an incidence angle of the radiation on the object θ = 45° were used as ATR elements. Recording was carried out in the wavenumber ($\tilde{\nu }$) range from 4000 to 500 cm^–1^, and the resulting spectra were plotted and processed using the OPUS software from Bruker. The proportion of hydrogen-bonded C=O groups in the urethane was estimated by deconvolution of the characteristic absorption bands using the OriginPro 2021 program (version 9.8.0.200, OriginLab Corporation, Northampton, MA, USA).

#### 2.3.2. NMR Spectroscopy

The chemical structure of segmented PUU was analyzed using an AVANCE II-500WB NMR-Fourier spectrometer (Bruker, Germany) using the TopSpin program version 3.5. ^1^H NMR spectra were recorded at an operating frequency of 400 MHz and a temperature of 298 K for samples previously dissolved in dimethyl sulfoxide (d^6^).

#### 2.3.3. X-ray Diffaction Analysis

The presence of long-range order in segmented PUU was revealed using the XRD method on a Rigaku SmartLab diffractometer (Tokyo, Japan). The registration was carried out in the range of angles 2θ from 10 to 40° at a scanning rate of 0.5° per minute. To calculate the average size of the coherent scattering regions formed in the PUU by the ordered soft PCL blocks having M_n_ = 2 × 10^3^, a profile analysis of XRD patterns was carried out in the PDWin 4.0 software package (NPP “Burevestnik”, Saint Petersburg, Russia) allowing one to determine the position of the XRD maxima and the full width of the peaks at half their height (FWHM). The average crystallite size for PUU was calculated using the obtained values of FWHM according to the Scherrer equation.

#### 2.3.4. Scanning Electron Microscopy

Scanning electron microscopy was performed on the different sides of the PUU films using a SUPRA-55VP microscope (Carl Zeiss, Oberkochen, Germany). The films were preliminarily etched for 20 min in a 0.5 M solution of sulfuric acid with KMnO_4_, after which they were thoroughly washed with distilled water and dried to a constant mass. The etched film pieces were fixed on microscope holders with special glue and covered with a thin layer of platinum.

#### 2.3.5. Polarizing Optical Microscopy

Melting of the crystalline phase of PUU using the composition S6 as an example was visualized with the help of a Linkam adjustable thermal stage of an AxioScope.A1 polarizing microscope (Carl Zeiss, Jena, Germany) at a magnification of ×40 with crossed beams at 90°. The heating of the sample film was carried out at the rate of 5°/min in the temperature range from 25 to 70 °C, holding every 5 °C for 3 min to achieve equilibrium by the multiblock copolymer.

#### 2.3.6. Differential Scanning Calorimetry

A differential scanning calorimeter DSC 204 F1 Phoenix (Netzsch, Bayern, Germany) was used to determine glass transition (*T*_g_), crystallization (*T*_cr_), and melting temperature (*T*_m_) of the synthesized PUU, which differ in the molecular mass of soft segments as well as in the chemical structure and length of hard aromatic blocks. Two temperature scans were performed for each sample under argon in the range from −80 to 250 °C. The choice of the upper temperature limit was due to the onset of thermal degradation of the PUU films above 250 °C; according to the data of thermogravimetric analysis (Figure S1, see Electronic Supplementary Materials—ESM). The heating rate of the samples during DSC experiments was 10 °C/min, while they were cooled twice as fast (20 °C/min) by means of liquid nitrogen. During heating and cooling as well as between temperature scans, the samples were not subjected to isothermal holding.

#### 2.3.7. Dynamic Mechanical Analysis

In the dynamic loading mode at the force of 0.1 N, a frequency of 1 Hz, a deformation amplitude of 0.1%, and a heating rate of 5 K/min, the films of synthesized PUU were tested on a DMA 242C setup (Netzsch, Germany). The DMA method was used to determine the moduli of accumulation of elastic deformation (*E*′) and mechanical losses (*E*′′). The mechanical loss angle tangent (tan *δ*) was determined by the Equation (1):

tan *δ* = *E*′′/*E*′ (1)

The obtained dependences tan *δ*(T) and *E*′(T) were used to determine the temperatures of relaxation processes in multiblock PUU.

#### 2.3.8. Small-Angle Neutron and X-ray Scattering Methods

The measurements of PUU films were carried out on a YuMO small-angle neutron scattering time-of-flight spectrometer equipped with two He^3^ ring detectors located at a distance of 4 and 13 m from the sample. Scattering intensity (differential cross section per sample volume) was recorded as a function of momentum transfer modulus *q* = (4π/*λ*)·sin(*θ*/2), where *θ* is the scattering angle and *λ* is the incident neutron wavelength. The distribution of the incident neutron beam provides a wavelength range from 0.5 to 8 Å, and a range of transmitted pulses from 0.006 to 0.6 Å^−1^. The typical data collection time was 50 min per sample.

Measurements of the X-ray scattering intensity were carried out on an automatic small-angle X-ray diffractometer “AMUR-K” with a single-coordinate position-sensitive detector OD3M at a fixed radiation wavelength *λ* = 0.1542 nm (CuKα line of a sharp-focus tube with a copper anode, pyrolytic graphite monochromator) and Kratky collimation system. The cross-section of the X-ray beam was 0.2 mm × 8 mm; the region of scattering angles, 2*θ*, corresponded to the range of the scattering vector modulus, *q*, from 0.01 up to 1.0 Å^−1^. The samples were placed in a vacuum chamber. The sample-detector distance was 700 mm. The measurement time for one sample was 10 min.

In both cases, the investigated polymer film (one layer) with a thickness of 60 μm was mounted on standard holders in such a way as to cover a hole of 14 or 3 mm for the neutrons or X-ray scattering, respectively. Additional SAXS measurements made it possible to refine the previously obtained SANS results, since the absence of incoherent scattering in SAXS and an increase in the range of the momentum transferred up to 1.0 Å^−1^ significantly improved the background component of the scattering curve that, in turn, led to a qualitative improvement in experiment data fitting by the model described in the corresponding section.

#### 2.3.9. Mechanical Testing

The study of the mechanical properties of the PUU films was carried out in the uniaxial stretching mode at a temperature of 23 °C and air humidity of 65% using an INSTRON 5943 universal tensile testing machine (USA) in accordance with ISO 527. The samples were tested at working dimensions parts 20 mm × 2 mm with a tensile rate of 100 mm/min. To measure the mechanical properties, at least seven samples from each series were tested. The following characteristics were determined from the test results: Young’s modulus (*E*), tensile strength (*σ_br_*), yield stress (*σ_y_*), and strain at break (*ε_br_*), which were calculated from the recorded stress-strain curves.

## 3. Results and Discussion

### 3.1. Chemical Structure and Hydrogen Bonding Interactions in PUU

**Figure 3:**
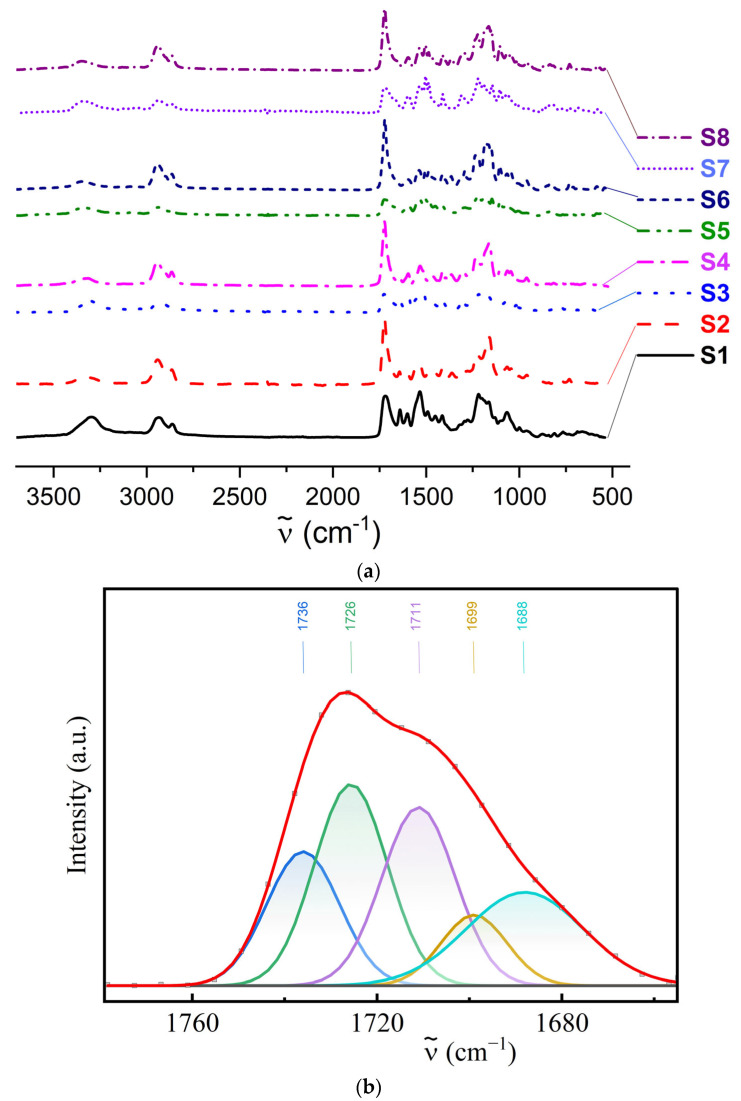

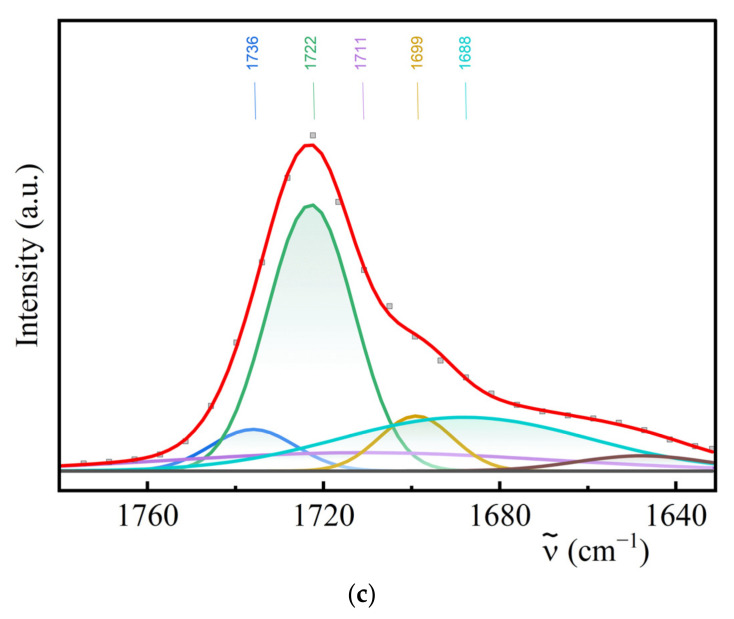
IR spectra for S1–S8 compositions of PUU (**a**) as the function of absorbance on wavenumbers. The deconvolution of the IR spectrum for S3 (**b**) and S8 (**c**) according to the methodology presented in [43]. The red line is the approximation using the six peaks of the Gaussian function [44]. (Figure S2, see Electronic Supplementary Materials—ESM).

The chemical structure of the synthesized PUU compositions was confirmed with the help of IR spectroscopy by correlating the identified absorption bands with vibrations of the corresponding functional groups and bonds. The absorption bands in the region of 3450–3400 cm^−1^ correspond to vibrations of free (non-hydrogen bonded) N–H of urethane and urea groups. The formation of hydrogen bonds by these groups can be judged using the absorbance, $A=\log_{10}\left(1/T\right)$, on the border of SWIR/MWIR regions (*ca*. 3 µm or 3350–3300 cm^−1^) of the IR spectrum (see Figure 3a).

Vibrations of free and H-bounded carbonyl groups of the urethane give absorption bands in the wavenumber ($\tilde{\nu }$) ranges 1730–1720 and 1720–1710 cm^−1^, respectively (Figure 3b,c). Moreover, ordered H-bonded C=O groups, which contribute to the formation of a certain order or regularity in the polymer, are characterized by a band at 1710 cm^-1^, while disordered ones (the regions remain amorphous) appear in the IR spectra at 1720 cm^-1^. As for the free C=O groups of urea, their vibrations attribute to absorption bands in the region of 1700–1690 cm^−1^. The bands of disordered and ordered H-bonded carbonyl groups of the urea are found at 1650 cm^−1^ and 1630 cm^−1^, respectively. Stretching vibrations of aromatic ring C=C bonds lie in the region of 1600–1500 cm^−1^. The ether group (C–O–C) bands are observed in the wavenumber range 1300–1000 cm^−1^. The remaining bands at 2940 and 2865 cm^−1^ belong to symmetric and asymmetric stretching vibrations of CH_2_ groups. The absence of an absorption band at 2265 cm^−1^ indicates that all isocyanate functional groups have reacted.

According to the IR spectra shown in Figure 3a, in all PUU obtained on the basis of PCL with M_n_ = 2 × 10^3^, the main portion of the N-H of urethane and urea groups is involved in the formation of the hydrogen bonds, as evidenced by the presence of the absorption bands with high intensity in the region of 3350–3300 cm^−1^ and low intensity in the range of 3450–3400 cm^-1^. In the case of multiblock copolymers synthesized using a shorter soft aliphatic segment, there are no bands characteristic of free N–H (Figure 3a). This means that all N–H of urethane and urea groups in these systems participate in the formation of hydrogen bonds with oxygen of similar groups in other units or neighboring chains.

According to the methodology proposed in [45], the proportion of H-bonded urethane groups (*C_b_*) was estimated (Table 1) using the following Equation (1):

(2) $$C_{b}=\frac{A_{1688}+A_{1699}}{k^{\prime }M_{PE}A_{1720}+A_{1688}+A_{1699}}$$

where *A*_1688_, *A*_1699,_ and *A*_1720_ are the areas of absorption bands of C=O vibrations of the urethane groups, and *k′* is a constant representing the ratio between the absorption coefficients of H-bonded and free urethane carbonyl groups, equal to 1.2. For this purpose, the C=O absorption bands of urethane groups first deconvoluted in the region of 1760–1640 cm^−1^. As a result, up to six components in the IR spectrum were separated in accordance with [45] (e.g., for S3 and S8 see Figure 3b,c). Peak maxima were at 1750, 1736, ca. 1725, 1711, 1699, and 1688 cm^−1^.

The *C_b_* value was compared with the fraction of the hard segments (HS) calculated by the Equation (3):

(3) $$C_{HS}=\frac{k\cdot M_{II}+l\cdot M_{CE}}{n\cdot M_{PE}+k\cdot M_{II}+l\cdot M_{CE}\;}100\%$$

where *C_HS_* is the mass fraction of hard segments, %; *k*:*l*:*n* is the molar ration equal to 2:1:1; the molecular masses of diisocyanate (*M_II_*), polyester (*M_PE_*), and chain extender (*M_CE_*), g·mol^−1^, are included.

According to the obtained results, it can be proved that the replacement of 2,4-TDI by 4,4′-MDI in the structure of hard PUU blocks has little effect on the change in the number of the H-bonded urethane groups (Table 1), whereas the inclusion of the diamine BAPS (Figure 1) into the structure of the multiblock copolymer as a chain extender leads to a significant increase (approximately 1.5–2 times) in the fraction of such bonds (*C_b_*). This leads to an essential rise in the fraction of H-bonded hard segments (*ϕ*) in the polymer mass (by a factor of 1.5–3.4). The use of soft aliphatic segments with a lower molecular mass contributes to a more efficient formation of the hydrogen bonds between C=O urethane groups in PUU. Reducing the length of PCL chains in the composition of multiblock copolymers results in a more frequent alternation of the urethane groups and an increase in the content of the H-bonded aromatic blocks: the fraction of the H-bonded HS (*ϕ*) increases by a factor of 2.3–5.8. However, the SANS data do not indicate enlargement of the hard phase domains; on the contrary, the latter’s sizes decrease. This result refers to microphase separation in the systems under investigation, the phase fraction of hard domain increasing, and d-spacing between them shortening concurrently with the PCL length.

**Figure 4:**
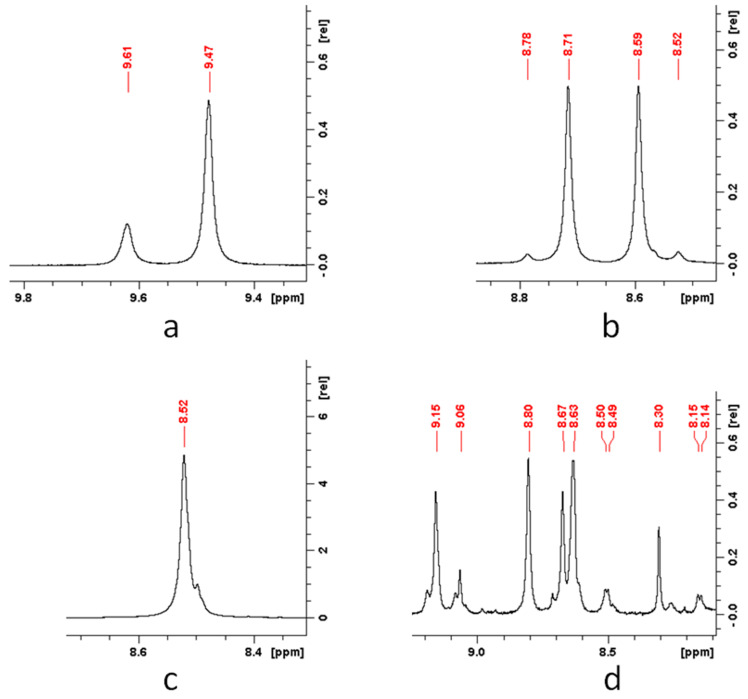
^1^H NMR spectra of model compounds: 4,4′-MDI-530PCL (**a**), 4,4′-MDI-BAPS (**b**), 4,4′-MDI-4,4′-MDI (**c**), 2,4-TDI-BAPS (**d**).

The chemical structure of the segmented PUU was also analyzed using nuclear magnetic resonance (NMR) spectroscopy. To interpret the proton NMR-spectra of the obtained multiblock copolymers, a number of model reactions between the monomers used to form the soft and hard segments were carried out (Figure S3, see Electronic Supplementary Materials—ESM). Copolymers based on 4,4′-MDI and PCL in a ratio of 1:1 were synthesized. The ^1^H NMR spectrum of the model system on an ester with M_n_ = 0.53 × 10^3^ contained chemical shifts of 9.62 and 9.48 ppm corresponding to the proton signals of the urethane groups (Figure 4a). When PCL with a higher molecular mass was used, the first peak was absent. This may indicate that some of the biodegradable polyester molecules with a molecular mass of 530 g·mol^−1^ have a structure obtained under conditions when the growth of the corresponding macromonomer occurred with the participation of only one hydroxyl group of diethylene glycol, the initiator of PCL polymerization, rather than two, as in the synthesis of aliphatic diol with M_n_ = 2 × 10^3^.

The next few model syntheses were carried out in order to describe the signals corresponding to the urea groups. In the first synthesis, 4,4′-MDI and diamine BAPS in a 1:1 ratio were used as monomers to obtain polyurea. The ^1^H NMR spectrum of this copolymer revealed chemical shifts in the region of 8.71 and 8.58 ppm, which are characteristic of the protons of the urea groups (Figure 4b). The second synthesis simulated a side reaction of the formation of oligomers from 4,4′-MDI molecules when water enters the reaction medium. The proton spectrum of the obtained product included only one signal in the region under consideration at 8.52 ppm and was characterized as a chemical shift of the protons of the side urea groups (Figure 4c). The third synthesis was carried out to describe the signals of the urea groups in multiblock copolymers obtained using 2,4-TDI. For this purpose, a reaction was carried out to obtain polyureas based on 2,4-TDI and diamine BAPS at a ratio of 1:1. During the analysis of the ^1^H NMR spectrum of polyurea, it was found that, due to the asymmetric structure of 2,4-TDI, about 10 signals appear in the region corresponding to the protons of the urea groups, the exact correlation with the structure of which is not possible (Figure 4d). Therefore, one can consider a successful synthesis proceeding only from the point of view of the presence or absence of a number of these characteristic signals.

**Figure 5:**
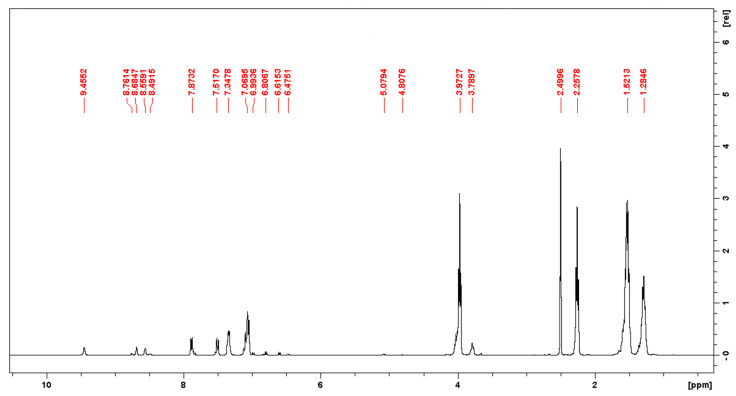
^1^H NMR spectrum of a multiblock PUU with composition S8.

Having become sure that the position of the chemical shifts of the protons of the target groups of urethane and urea in the ^1^H NMR spectra of the model systems and the synthesized PUU coincided, the remaining signals were identified. Chemical shifts lying in the region from 8 to 7 ppm were correlated with the protons of aromatic rings in the structure of the hard segments of the PUU (Figure 5). In addition, in the range from 7 to 6 ppm, there may be signals that coincide in position with the chemical shifts of the protons of the aromatic rings of the pure monomers. Since in the case of PUU synthesized on 4,4′-MDI, their intensity correlated with the intensity of chemical shifts by 8.79 and 8.51 ppm located near the signals of the target urea groups, it was assumed that these were protons of the terminal units of the polymer chain. This assumption is also supported by the presence of signals in the region of 5.1–5.0 and 4.87 ppm. Their location coincides with the chemical shifts of the NH_2_ groups of the chain extender and amino groups formed by the interaction of 4,4′-MDI with water, and the intensity coincides with the intensity of the two groups of signals described above. Signals in the area 4.1–4.0 ppm were attributed to the proton signals of the methylene groups located next to the ester groups in the PCL structure. Chemical shifts at 3.79 and 3.67 ppm are present only in the spectra of multiblock copolymers obtained using 4,4′-MDI and belong to the protons of the methylene groups in the structure of the corresponding diisocyanate. The ^1^H NMR spectra of TDI-based PUU show chemical shifts of 2.2–2.1 ppm, which correspond to the protons of the methyl groups in the structure of the asymmetric diisocyanate. For chemical shifts in the region of 1.5–1.2 ppm, the protons of the methylene groups in the PCL structure are responsible.

### 3.2. Relaxation and Phase Transitions in Multiblock PUU

**Figure 6:**
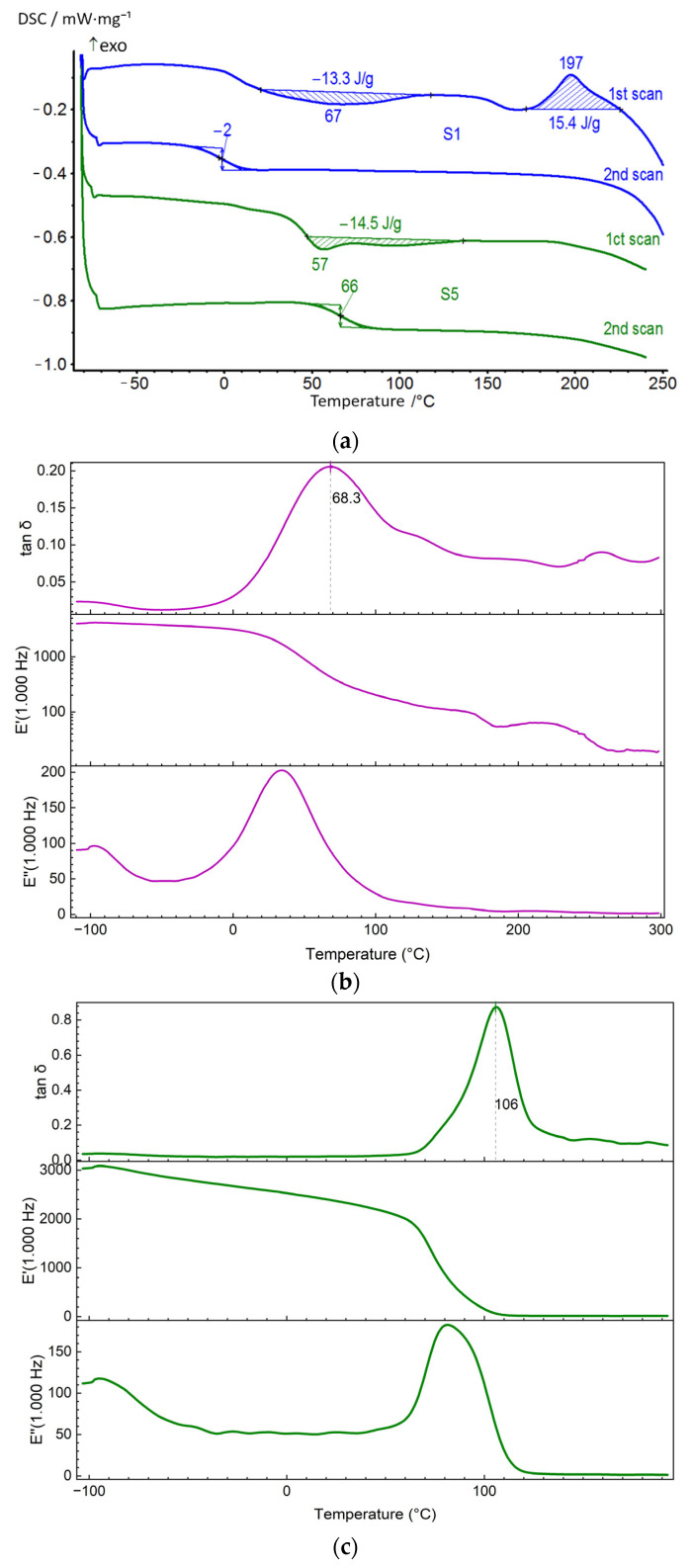
DSC curves (**a**) of the segmented PUU for S1 and S5 and their DMA (**b**,**c**), respectively.

**Figure 7:**
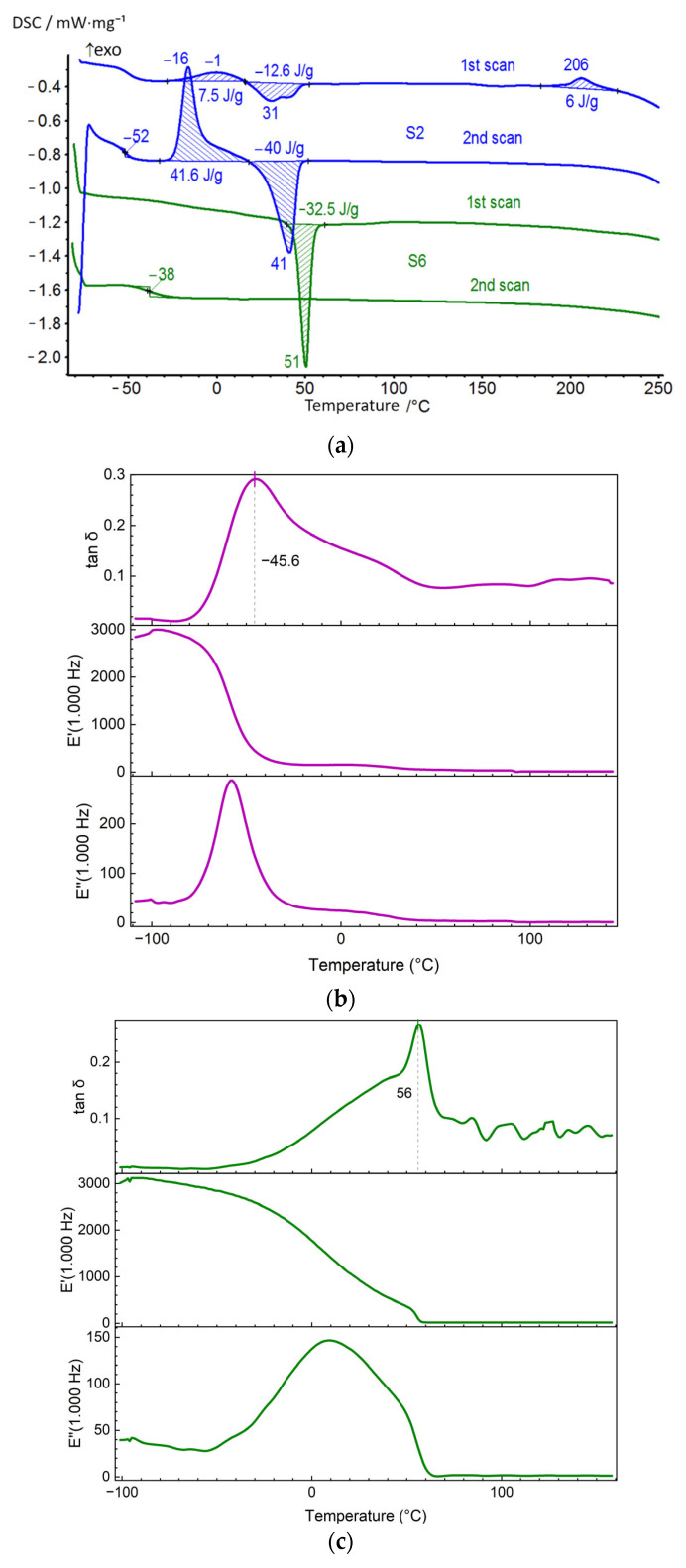
DSC curves (**a**) of segmented PUU for S2 and S6 and DMA (**b**,**c**), respectively.

For the synthesized PUU, the effect of the molecular mass of soft segments as well as the length and structure of hard blocks on the temperatures of relaxation and phase transitions were estimated using DSC. It was shown that the use of PCL as the soft segments with a molecular mass of 2000 instead of 530 g·mol^−1^ contributes to a decrease in the glass transition temperature of multiblock PUU, regardless of the chemical structure of their hard blocks (Table 2). Thus, according to the DSC curves obtained during the second scan, the *T_g_* value of mPDA-based PUU decreases by *ca*. 50 °C when moving from S1 to S2 as well as from S3 to S4 (Figure 6a and Figure 7a, *cp*. S1 and S2). Comparative analysis of PUU having the same molecular mass of the soft segments and different lengths of hard blocks showed that the use of 4,4′-MDI instead of 2,4-TDI in their synthesis had little effect on the thermal activation of the PCL segmental mobility and an increase in the free volume fraction in the multiblock copolymers upon heating. The inclusion of diamine BAPS as a chain extender in the PUU structure contributes to a significant increase in the transition temperature from the glassy to the highly elastic state compared to similar compositions based on mPDA (S1–S4).

**Figure 8:**
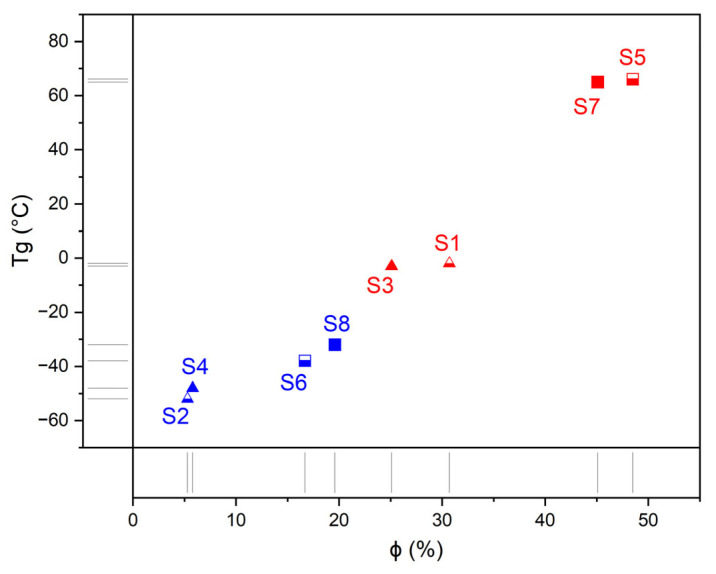
Correlation between the glass transition temperature (*T*_g_, Table 2, @ 2nd scans) in PUU S1–S8 and the fraction of bonded hard segments (*ϕ* value, Table 1). PUU are based on PCL with a molecular mass of 530 (red) or 2000 g·mol^−1^ (blue), on mPDA (triangles) or BAPS (squares), and on TDI (half solid) or MDI (solid).

It is noteworthy that the *T*_g_ for S5 and S7 decreases by 100 K compared to S6 and S8, respectively (Table 2, Figure 8, @ 2nd scans). Moreover, the difference Δ*T*_g_ is twice as large as for S1 (S3) relative to S2 (S4), where the Δ*T*_g_ value is only 50 K. In other words, the longer soft PCL segments (M_n_ = 2 × 10^3^) more effectively disrupt the interaction between long hard blocks (BAPS) than between short ones (mPDA). This fact can be explained by the presence of a more energetic interaction between the long HS containing BAPS than between the short ones containing mPDA. It can be assumed that the enhanced interaction between the hard segments is due to the hydrogen bonding of sulfone and urea groups, which are absent in the mPDA HS, in addition to the urethane and urea interaction. This hypothesis is confirmed by IR spectroscopy data, from which the fractions of bonded hard segments, *ϕ*, in multiblock PUU by the interaction of urethane groups were obtained (Table 1): the value of *ϕ* is positively correlated with *T*_g_ (Figure 8).

For most of the compositions of the synthesized PUU, exo- and endothermic effects are observed on the DSC curves during the first scan, which are associated with the structuring and disordering of soft and hard segments upon heating. In the multiblock copolymers S1, S3, S5, and S7, the film formation and solvent removal result in the ordering of a small fraction of the soft PCL blocks with M_n_ = 0.53 × 10^3^. Their disordering is evidenced by the time-stretched endothermic effects on the DSC curves of the above PUU compositions in the temperature range from 20 to 135 °C (Figure 6a and Figure 7a). In addition, for multiblock copolymers based on mPDA (S1, S3), an exothermic effect appears at about 200 °C, associated with additional structuring of the aromatic blocks at high temperatures. According to thermogravimetry data, thermal degradation of the samples starts above 250 °C, and the temperature at which the sample loses 5% of its mass (*τ_5_*) lies in the range from 260 to 300 °C (Figure S1, see Electronic Supplementary Materials—ESM). Therefore, it is not possible to trace the thermal effect corresponding to the disordering of the domains structured on the base of TDI- and MDI-mPDA hard blocks in the PUU. In the case of multiblock copolymers of the composition S5 and S7, the ordering of hard blocks up to 250 °C does not occur because of the large length of the chain extender. An additional factor may be the formation of hydrogen bonds between sulfone and urea groups, which hinders the kinetic units and leads to the inability to order on the second annealing cycle. Cooling (20 °C/min) PUU S1, S3, S5, and S7 down to −80 °C and reheating them (10 °C/min) up to 250 °C in the DSC mode led to film amorphization, which made it possible to record only the transition from glassy to highly elastic state for these compositions during the second scanning (Figure 6a and Figure 7a).

If one considers a series of PUU based on PCL with M_n_ = 2 × 10^3^, then on the DSC curves, in addition to the relaxation process, phase transitions with large thermal effects in soft segments are observed during the first scan compared to the systems S1, S3, S5, and S7 (Table 2). A weak exothermic effect at 206 °C corresponding to the ordering of the hard blocks was found only for S2, where the TDI-mPDA units have the highest mobility (Figure 7a). According to the DSC curves obtained during the first scan, the S2, S4, S6, and S8 are initially semi-crystalline multiblock copolymers. Upon secondary heating, the soft segments in S2 and S4 tend to self-order above *T_g_*, and *T*_cr_ increases with the length of the hard blocks in the series TDI-mPDA → MDI-mPDA. This is not observed for S6 and S8 because of the hindrance due to the length of HS and hydrogen bonding by sulfone groups. The exothermic effect of the soft blocks structuring on the DSC curves recorded for the samples S2 and S4 is followed by a process accompanied by heat absorption, which refers to the disordering of the formed crystalline PCL domain. Moreover, the exo- and endothermic effects not only follow each other on the temperature scale but are also identical in their magnitude. That is, during the second scan, the soft segments of the PUU possessing a short length of the hard blocks, have time to arrange themselves and after that, they melt upon further heating. In PUU based on diamine BAPS (S6, S8), the PCL chains do not recrystallize after the second scan, since their mobility is limited by the massive hard blocks (Table 2, Figure 7a).

In DSC, *T*_g_ is determined from the measured heat flux, while the DMA method is sensitive to mechanical relaxation. Therefore, dynamic experiments (DMA) show higher glass transition temperatures than a static approach (DSC). The DMA method, which is sensitive to relaxation processes, despite the difference with DSC in the values of the glass transition temperature (α-relaxation), does not contradict the previously revealed trends. Peaks are observed on the curves of tan *δ*–*T* at about −100 °C, 50 °C, 120 °C, and 190 °C. They are referred to as γ, α_S_, α_H,_ and δ, respectively (Table 3) [46]. The relaxation α_S_, α_H_ -transition could be attributed to the devitrification of the SS and HS domain in PUU. However, glass transition is a more complex process, which includes molecular-kinetic and structural aspects related to the change in free volume. Therefore, one could say that in this case, the activation of cooperative modes of segmental mobility occurs, i.e., mobility is unfrozen. Comparison of the α_H_-transition temperature, *T*_α__H_, determined by DMA for the synthesized PUU made it possible to relate the increase in *T*_αH_ to the fraction of hard segments, *C****_HS_***, in the multiblock copolymer structure (Table 1and Table 3). It is shown that, with the same structure of the hard blocks, the inclusion of PCL with a higher molecular mass in the PUU structure leads to a decrease in the temperatures of the α_H_, α_S_-transition. Thus, *T*_αS_, *T*_αH_ determined from the mechanical loss (tan *δ*) is, on average, 100 K higher for S1 and S3 as compared to similar multiblock copolymers based on PCL with M_n_ = 2 × 10^3^ (S2 and S4). The use of diamine BAPS instead of mPDA as a chain extender at a fixed length of soft segments also contributes to a higher *T*_αS_, *T*_αH_ value.

For the amorphous part of multiblock copolymers, in addition to the transition from the glassy to the highly elastic state, DMA can also be utilized to observe several small-scale relaxation processes, often called additional relaxations [46,47]. Thus, for all PUU, the temperature dependences of tan *δ* and *E*″ show the peak at ca. −90 °C, accompanied by a change in the slope on the *E*′(*T*) curves. This peak corresponds to the movement of the methylene group –CH_2_– in the composition of the soft PCL blocks (*γ*-relaxation). In addition, on the curves of the mechanical loss, shoulders in the left region of maximum were observed, which indicates a possible β-relaxation process. The β-transition could be associated with the unfreezing of the mobility of the soft segments in limited regions of low density. However, these additional relaxations are extremely weak, and they cannot be reliably established for all compositions of the synthesized PUU.

It should be noted that, according to the DMA data, the S1 and S3 do not have a plateau of rubber-like elasticity, at which *E*’ is practically independent of the temperature. The use of a diamine BAPS with the same length as the soft aliphatic segments leads to the formation of thermoplastic elastomers with the high-temperature plateau.

### 3.3. Structural and Morphological Features of Multiblock PUU

**Figure 9:**
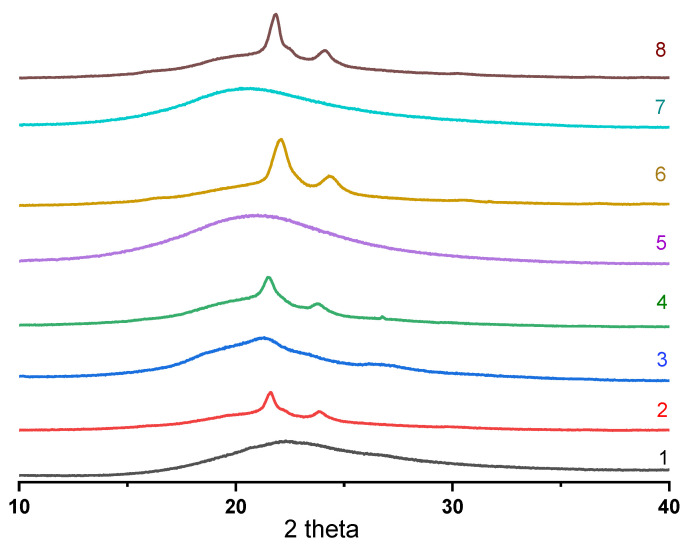
X-ray diffraction patterns of segmented PUU: S1 (**1**), S2 (**2**), S3 (**3**), S4 (**4**), S5 (**5**), S6 (**6**), S7 (**7**), and S8 (**8**).

As with the DSC, the XRD method showed that the initial PUU films with long PCL segments formed on a glass substrate have the structure of a semi-crystalline polymer, regardless of the structure of the hard aromatic blocks. Their XRD patterns in the region 2*θ* = 15–30° show sharp peaks at Bragg angles of 21.4° and 23.7°, which are attributed to the crystal planes of (110) and (200) and indicate the existence of the orthorhombic crystal lattice of semi-crystalline PCL in PUU [48,49]. In the case of the same compositions of multiblock copolymers based on PCL with M_n_ = 0.53 × 10^3^, only a wide amorphous halo was observed in this angle range since the length of the soft aliphatic segments with sufficiently massive aromatic blocks is insufficient for self-ordering (Figure 9). The average size of the coherent scattering regions, being calculated according to the Scherrer formula, varied from 10 to 17 nm for the structured part of PCL with M_n_ = 2 × 10^3^ in the synthesized multiblock copolymers, depending on the structure.

**Figure 10:**
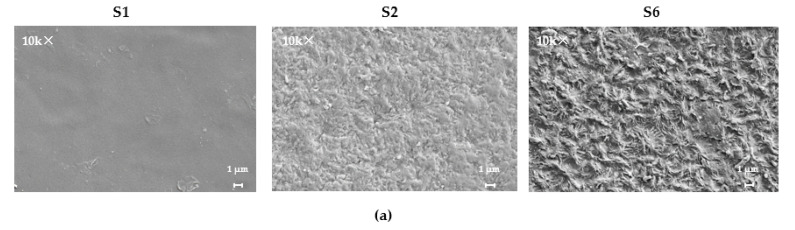

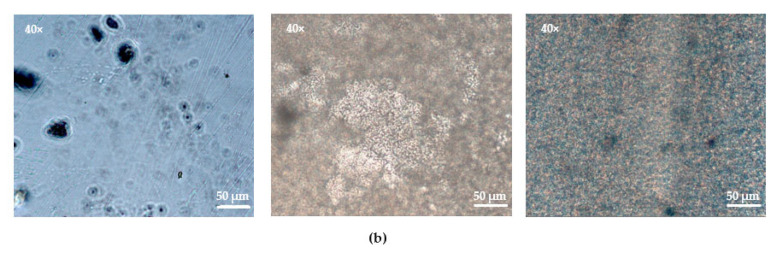
SEM micrographs (**a**) and POM images (**b**) of segmented PUU of the compositions S1, S2, and S6.

SEM micrographs confirm the presence of a crystalline phase formed due to the ordering of the soft segments in the form of spherulites several microns in size for PUU compositions based on PCL with M_n_ = 2 × 10^3^. (Figure 10a, micrographs 2, 3). In the samples S1, S3, S5, and S7, nothing similar is observed because the length and molecular mobility of the aliphatic segments bounded by hard-phase domains are insufficient for self-ordering (Figure 10a, micrographs 1). It is noteworthy that, according to SEM data, the proportion of spherulites on the surface of the etched PUU films is higher for compositions S6 and S8 in comparison with their analogs including mPDA as a chain extender (Figure 10a, *cp*. S2 and S6). The same pattern can be observed using a polarized optical microscope (Figure 10b). For the sample S1, a completely amorphous structure is diagnosed; upon transition to a similar composition of PUU having longer soft segments (S2), one can reveal islands of the crystalline phase formed due to the ordering of PCL chains with M_n_ = 2 × 10^3^. A fully crystalline field is observed in the crossed beams of a polarized optical microscope (POM) only for the S6-composition of PUU. Thus, along with DSC and XRD, SEM and POM were able to confirm the presence of a crystalline phase formed by SS in PUU S2, S4, S6, and S8 as well as visualize crystal regions in these multiblock copolymers.

**Figure 11:**
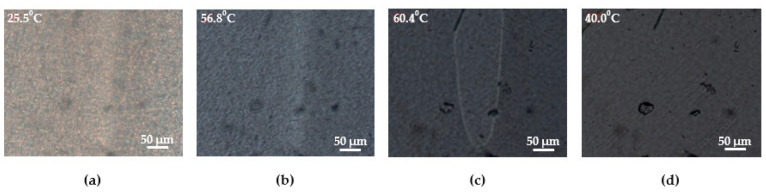
POM micrographs of the multiblock PUU of the composition S6 made at different temperatures: 25.5 (**a**), 56.8 (**b**), 60.4 (**c**), and 40.0 °C (**d**).

To visualize the phase transitions in segmented PUU, the S6 film was heated on a POM thermal stage from 25 up to 70 °C, followed by its slow cooling to room temperature while fixing images of the corresponding thermodynamic states (Figure 11). The melting of the crystalline phase formed by the soft segments began at about 57 °C, which practically coincides with the T_m_ determined using DSC (Figure 11b,c). Upon cooling of the PUU film below T_m_, it did not recrystallize (Figure 11d), which also does not contradict the DSC data.

As for the sizes of the regions where the hard blocks are concentrated (the hard phase domains), they were estimated using the SANS and SAXS methods. To approximate the small-angle scattering data, the model of the broad Lorentzian-type peak at the end of the power-law decay appeared to be of advantage with SasView software [50]. (See Figure S4 and Section S4 in Electronic Supplementary Materials—ESM) At that, the scattering intensity I(q) is calculated as:

(4) $$I\left(q\right)=\frac{A}{q^{n}}+\frac{C}{1+{\left(\left|q-q_{0}\right|\xi \right)}^{m}}+B\;$$

Here the peak position is related to the *d*-spacing as $q_{0}=2\pi /d_{0}$. A is the Porod law scale factor; n is the Porod exponent; C is the Lorentzian scale factor; m is the exponent of q; ξ is the screening length; and B is the flat background. The *d*-spacing corresponding to the broad peak is a characteristic distance between the scattering inhomogeneities in the segmented polymeric system under investigation.

Since an increase in the length of the aromatic blocks leads to an increase in the values of ξ (the hard domain characteristic radius) and the *d*-spacing (the distance between hard domains) at a fixed length of the aliphatic segments, one can state with some certainty that the hard phase domains act as scattering centers. The data of SANS and SAXS agree well and show an increase in the hard phase domain size (2ξ) from 28 to 68 Å with an increase in the PUU aromatic block length in the series of 2,4-TDI + mPDA → 4,4′-MDI + mPDA → 2,4-TDI + BAPS. If one compares multiblock copolymers with different molecular masses of soft aliphatic segments with an identical structure of aromatic blocks, then in PUU based on PCL with M_n_ = 2 × 10^3^ the distance between the domains of the hard phase will naturally be greater (Table 4).

**Figure 12:**
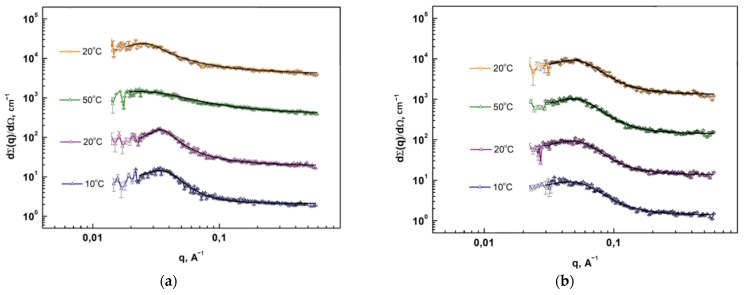
Dependences of the neutron scattering cross section on the momentum transfer for different stages during the temperature experiment for S6 (**a**) and S4 (**b**). The solid black lines are approximation according to Equation (4).

It should be noted that according to SANS data for the S6 heated at 10, 20, and 50 °C and then cooled to room temperature, partial restoration of the domain structure is observed (Figure 12a). Apparently, as a result of the disordering of the soft segments nearby their melting temperature, *T*_m_, the system of hydrogen bonds in the hard phase domains is broken, and asymmetric diisocyanates and massive BAPS diamines prevent the restoration of intermolecular interactions between the aromatic blocks. In the case when the hard segments are formed by 4,4′-MDI and mPDA, isothermal exposure of PUU above the PCL value of T_m_ does not affect the domain size and the distance between the scattering centers, which may indicate strong intermolecular interactions in the hard phase domains (Figure 12b).

### 3.4. Deformation-Strength and Thermomechanical Properties of PUU

**Figure 13:**
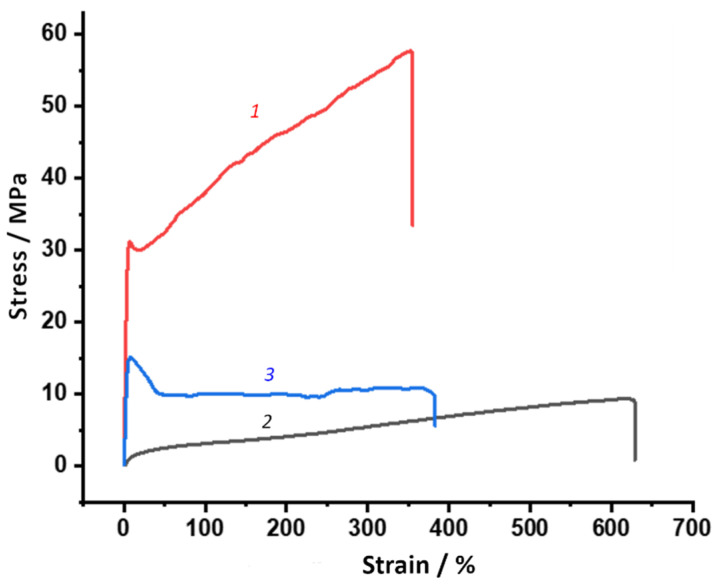
Strain-stress curves for S1 (*1*), S2 (*2*), and S6 (*3*).

Measurements of the mechanical characteristics of the PUU films in the uniaxial stretching mode showed that the compositions based on PCL with M_n_ = 0.53 × 10^3^ exhibit the properties of thermoplastics. They are characterized by high values of elasticity modulus and tensile strength in comparison with multiblock elastomers, which include PCL with M_n_ = 2 × 10^3^ in their composition. The ε_br_, on the contrary, in the case of S1, S3, S5, and S7, is significantly lower than that of elastomers of a similar structure (Table 5). In addition, for the PUU with shorter soft aliphatic segments, the presence of a yield point (σ_y_) is found, and a hardening stage is observed on the stress-strain curves (Figure 13). 

As confirmed by DSC and XRD data, the presence of ordered PCL segments in the structure of PUU for S2, S4, S6, and S8 should lead to toughening and embrittlement of multiblock copolymers. However, in contrast to this, a significant decrease in both free and hydrogen-bonded HS due to the high M_n_ aliphatic chains overrides this effect and imparts elastic properties to the examined systems. If you compare the segmented PUU with 4,4′-MDI in the hard block structure with the analogous multiblock copolymers containing 2,4-TDI in the structure, the elastic modulus values of the latter ones are higher. Since the share of hydrogen-bonded hard segments is approximately equal for the pairs S1 and S3, S2 and S4, S5 and S7, and S6 and S8, this may be due to a denser packing of aromatic blocks containing 2,4-TDI in the hard phase domains. This is supported by the SAXS data comparing S1 and S3 as well as S2 and S4 compositions. Deformation hardening is typical for all PUU synthesized on the base of diamine BAPS due to an increase in the proportion of hard segments and the number of hydrogen-bonded aromatic blocks as well as the enlargement of hard phase domains (Figure 13, Table 5). When replacing mPDA with diamine BAPS in the PUU with mechanical properties typical for elastomers, the systems are obtained, which occupy an intermediate position between thermoplastics and rubber. As compared to mPDA, diamine BAPS considerably increases *E* and σ_br_ in multiblock PUU while reducing the strain at break. Considering the rubber-like elasticity plateau of S6 and S8 recorded by DMA, it is possible to refer these multiblock copolymers to the class of thermoplastic elastomers.

**Figure 14:**
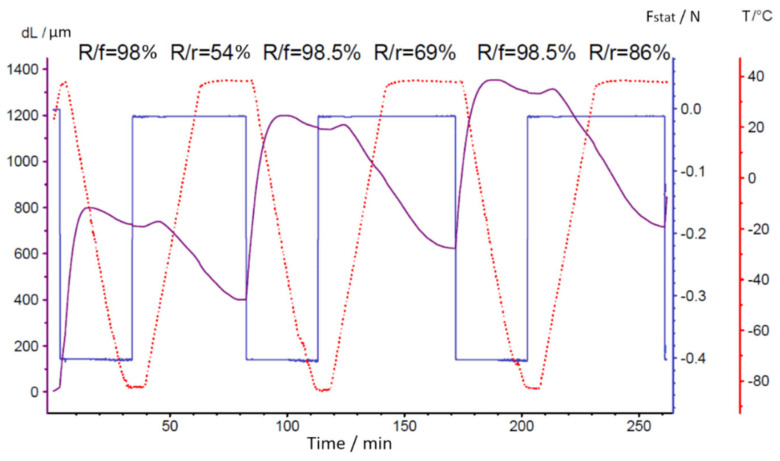
Cyclic thermomechanical tests of S2-segmented PUU in DMA mode.

The presence of the shape memory effect in PUU was determined in the DMA mode with an applied static force of 0.1 N in the temperature range of −120…+100 °C, the boundaries of which were set on the basis of the phase transitions recorded by the DSC method. The tested PUU compositions in the first thermomechanical cycle showed acceptable values (98%) of the shape fixation coefficient (*R_f_*) and low shape recovery coefficients (*R_r_*) (Table S1, see Electronic Supplementary Materials—ESM). Using the S2 as an example, it was revealed that thermomechanical training of the PUU samples contributes to a gradual increase in the *R_r_* value, probably due to the removal of mechanical stresses arising during the film formation and the approach of the multiblock copolymer structure to an equilibrium state (Figure 14).

## 4. Conclusions

Segmented copolymers are of great expectation in connection with the development of shape memory materials. In the present work, we tried to show the relationship between the thermal as well as mechanical properties of new multiblock polyurethanes and the “primary” structure (molecular mass of the soft segments) as well as the chemical structure of the hard segments. The flexibility of the segmented macromolecules is determined by the ratio of “soft” and “hard” chain parts in them. Indeed, an increase in the PCL segment length leads to a decrease in the glass transition temperature. In addition, increasing the length of the hard (aromatic) blocks causes a rise in *T*_g_. The revealed relationships between the chemical structure of monomeric units, the processes of self-organization of segments of different nature in the main chain, the domain structure formation, and relaxation and phase transitions will finally make it possible to give a clearer approach to the scientific recommendations for purposeful preparation of materials with predictable thermomechanical behavior. Further studies of these block copolymers can be related to establishing more precise connections between their mechanical properties and the relaxation spectra and to the comparison of the mobility of various morphological structures with mechanical relaxation times.

## Figures and Tables

**Table 1:** Results of calculating the fraction of H-bonded urethane groups in multiblock PUU.

Composition	Hard Segment		M_n_ PCL, g·mol^−1^	Fraction of Hard Segments, *C_HS_*, %	Fraction of H-Bonded Urethane Groups, *C_b_*, %	Fraction of Bonded Hard Segments, *ϕ* ^(1)^, %
	Group of I Stage	Group of II Stage				
S1	TDI	mPDA	530	46.3	66.3	30.7
S2	TDI	mPDA	2000	18.6	28.3	5.3
S3	MDI	mPDA	530	53.5	47.0	25.1
S4	MDI	mPDA	2000	23.3	25.1	5.8
S5	TDI	BAPS	530	59.6	81.4	48.5
S6	TDI	BAPS	2000	28.1	59.4	16.7
S7	MDI	BAPS	530	63.8	70.7	45.1
S8	MDI	BAPS	2000	31.8	61.7	19.6

Note: ^(1)^ the fraction of bonded hard segments, *ϕ = C_HS_·C_b_*, was estimated.

**Table 2:** Thermal properties of segmented PUU.

Composition	Scan Number	Thermal Characteristics of the Films				
		T_g_, °C	T_cr_, °C	ΔH_cr_, J/g	T_m_, °C	ΔH_m_, J/g
S1	1	8	197	15.4	67	−13.3
	2	−2	-	-	-	-
S2	1	−49	−1; 206	7.5; 6	31	−12.6
	2	−52	−16	41.6	41	−40
S3	1	5	203	2.5	58	−5.4
	2	−3	-	-	-	-
S4	1	−44	-	-	46	−34.2
	2	−48	−11	10.6	43	−42.4
S5	1	11	-	-	57	−14.5
	2	66	-	-	-	-
S6	1	-	-	-	51	−32.5
	2	−38	-	-	-	-
S7	1	54	-	-	97	−3.2
	2	65	-	-	-	-
S8	1	-	-	-	45	-7
	2	−32	-	-	-	-

**Table 3:** Thermomechanical properties of segmented PUU.

Composition	DMA Characteristics					
	T_γ_, °C	T_β_, °C	T_αS_, °C	T_αH_, °C	T_δ_, °C	Plateau (E′), °C
S1	−98	34	68	130	205	–
S2	−95	−58	−45	29	90	100–140
S3	−74	28	47	160	255	–
S4	−60	–	−48	20	117	50–80
S5	−90	50	106	155	–	130–190
S6	−95	15	56	80	–	60–160
S7	−96	50	108	178	219	140–230
S8	−98	−14	55	125	–	65–160

**Table 4:** Structural parameters of segmented PUU determined by the SANS and SAXS methods.

Composition	Method	ξ, Å	*d*-Spacing, Å
S1	SANS	14 ± 2	63
	SAXS	13 ± 1	60
S2	SANS	26 ± 5	78
	SAXS	17 ± 1	74
S3	SANS	25 ± 3	94
	SAXS	26 ± 1	100
S4	SANS	28 ± 4	134
	SAXS	28 ± 4	119
S6	SANS	35 ± 15	299
		

**Table 5:** Mechanical characteristics of the segmented PUUs films.

Composition	Films Properties			
	E, MPa	σ_br_, MPa	σ_y_, MPa	ε_br_, %
S1	995 ± 74	43 ± 14	44 ± 7	339 ± 135
S2	14 ± 1	9 ± 1	-	664 ± 79
S3	488 ± 37	48 ± 7	48 ± 6	196 ± 85
S4	4 ± 1	27 ± 3	-	861 ± 115
S5	1732 ± 113	66 ± 11	74 ± 3	266 ± 83
S6	535 ± 25	11 ± 1	16 ± 1	391 ± 141
S7	1721 ± 162	85 ± 12	75 ± 4	301 ± 75
S8	311 ± 26	27 ± 5	11 ± 1	1043 ± 166

## Data Availability

The data presented in this study are available on request from the corresponding author.

## Supplementary Materials

The following supporting information can be downloaded at: https://www.mdpi.com/article/10.3390/polym14194145/s1, Figure S1. TGA curves of PUU: S1, S2, S5, and S6.; Figure S2. Unfolded infrared spectrum for S3 (MDI-530PCL-MDI-mPDA) (a) and S8 (MDI-2000PCL-MDI-BAPS) (b). The red line is an approxi-mation using the six peaks of the Gaussian function [1,2].; Figure S3. 1H NMR spectra of model compounds: 4,4ʹ-MDI-530PCL (a), 4,4ʹ-MDI-BAPS (b), 4,4ʹ-MDI-4,4ʹ-MDI (c), 2,4-TDI-BAPS (d).; Figure S4. Parameters of fitting according the model (Equation S2) (a). The SANS data for S2 (TDI-2000PCL-TDI-mPDA) (b). The orange curve is the fitting upon Equation S2. The residuals (normalized) for fitting according the model (Equation S2) (c).; Table S1. Shape memory properties of the segmented PUU.
