# Supplementary material for: Domain Structure, Thermal and Mechanical Properties of Polycaprolactone-Based Multiblock Polyurethane-Ureas under Control of Hard and Soft Segment Lengths
Source: Polymers (Basel). 2022 Oct 3;14(19):4145. doi: 10.3390/polym14194145 (PMC9571805; doi:10.3390/polym14194145)
Supplement: Supplementary file 1 [file polymers-14-04145-s001.zip › polymers-1907233 supplementary.pdf]

Electronic Supplementary Materials.

Domain structure, thermal and mechanical properties of polycaprolactone-based multiblock poly(urethane-urea)s under control of hard and soft segment lengths

Alexander N. Bugrov [1,2,3]*, Yulia E. Gorshkova [4], Elena M. Ivan'kova [1,*], Gennady P. Kopitsa [5], Alina A. Pavlova [5], Elena N. Popova [1], Valentina E. Smirnova [1], Ruslan Y. Smyslov [1,5], Valentin M. Svetlichnyi [1], Gleb V. Vaganov [1] and Boris V. Vasil'ev [1,2,3]

1 Institute of Macromolecular Compounds of Russian Academy of Sciences, V.O., Bol'shoy pr. 31, 199004 St. Petersburg, Russia
2 Department of Physical Chemistry, Saint Petersburg Electrotechnical University (ETU "LETI"), str. Professora Popova 5, 197376 St. Petersburg, Russia
3 Ioffe Institute, str. Politechnicheskaya 26, 194021 St. Petersburg, Russia
4 Joint Institute for Nuclear Research, Joliot-Curie 6, 141980 Dubna, Moscow Region, Russia
5 Petersburg Nuclear Physics Institute NRC KI, 188300 Gatchina, Leningrad Region, Russia
* Correspondence: anbugrov@etu.ru (A.N.B.); ivelen@mail.ru (E.M.I.); Tel.: +7-(812)-323-6269 (A.N.B.)

S1. Thermogravimetric analysis

The choice of the upper temperature limit was due to the onset of thermal degradation of the PUU films above 250 °C according to the data of thermogravimetric analysis (Figure S1).

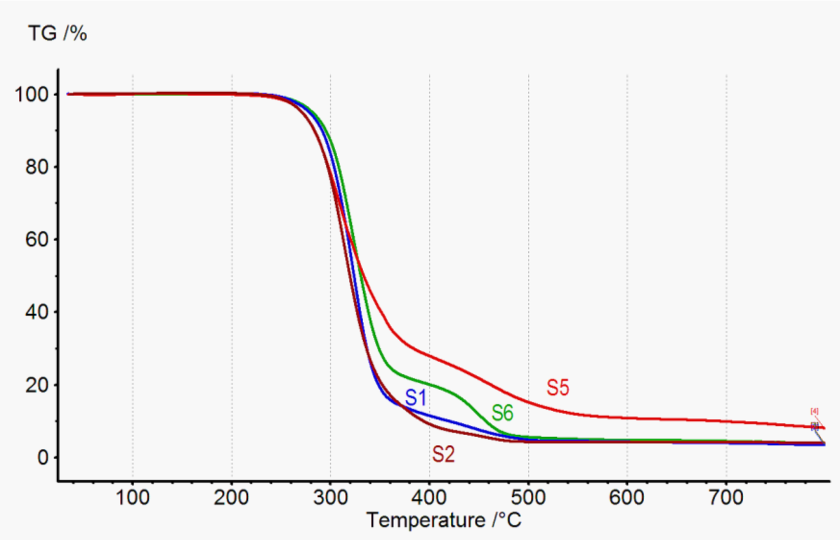

Figure S1. TGA curves of PUU: S1, S2, S5, and S6.

S2. Infrared spectroscopy

We observe the 1740–1690 cm^{-1} band for the urethanes R-O-CO-NH-R′. The maximum absorption intensity is observed in the IR spectra at 1720 cm^{-1} for monosubstituted derivatives of the urethane group (Figure S2).

Niemczyk [45] proposed a new definition of hydrogen bonding and dipole interaction and calculated the degree of microphase separation (DMS) for polycarbonate-based PU. He divided the -C=O peak into six peaks: free -C=O from carbonate segments manifested at about 1750 cm^{-1}, dipole -C=O in SS at about 1736 cm^{-1}, free -C=O for HS manifested at about 1720 cm^{-1}, hydrogen-bonded -C=O group of carbonate (with urethane groups) gave signal at about 1711 cm^{-1}, The hydrogen-bonded -C=O for urethane groups constituting the disordered and ordered and disordered solid phase is represented by peaks at about 1699 cm^{-1} and 1688 cm^{-1}, respectively. In addition, the content of hydrogen-bonded urethane groups (C_b) can be calculated according to the equation:

$$C_b = \frac{A_{1688}+A_{1699}}{k' M_{PE} A_{1720}+A_{1688}+A_{1699}}, \quad \text{(S1)}$$

where S_{1688}, S_{1699} and S_{1720} are the areas of the corresponding bonding peak, and k′ is a constant equal to 1.2 and represents the ratio between the absorption coefficients of hydrogen bonding and free urethane carbonyl groups.

a

Peak Analysis

Data Set:[MDIPCL530MF25]"Input Data"!B"Ired" Date:24.08.2022
BaseLine:Constant
Chi^2=2.58085E-06 Adj. R-Square=9.99857E-01 # of Data Points=65
SS=1.29042E-04 Degrees of Freedom=50

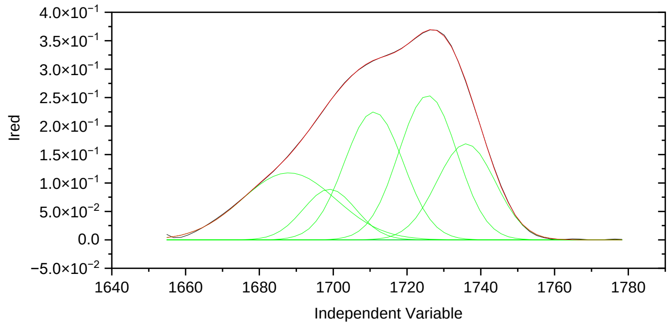

Fitting Results

Peak Index	Peak Type	Area Intg	Area IntgP	Center Grvty	Max Height	FWHM
1	Gaussian	0	0	1750	0.01004	0.04678
2	Gaussian	−3.35325	−18.43721	1736	0.16879	18.66315
3	Gaussian	−5.02047	−27.60411	1725.65865	0.25377	18.58529
4	Gaussian	−4.48651	−24.66822	1711	0.22478	18.7504
5	Gaussian	−1.59198	−8.75321	1699	0.08886	16.83083
6	Gaussian	−3.73519	−20.53725	1688	0.11779	29.9282
7	Gaussian	0	0	1650	0.5109	0.12258

b

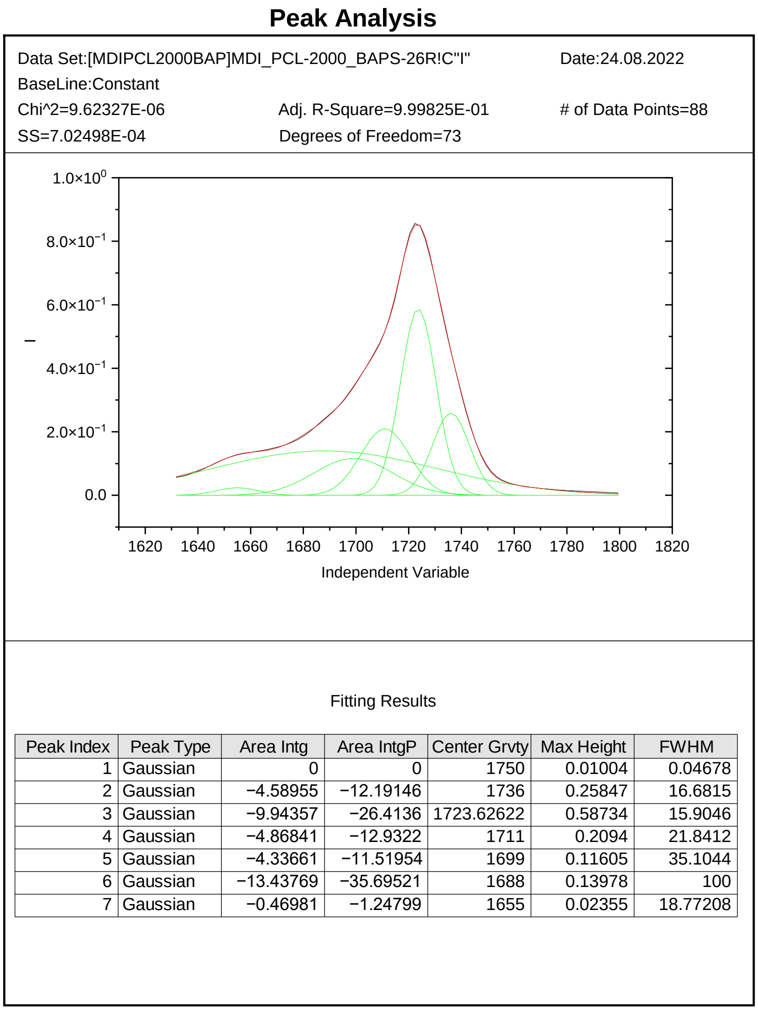

Peak Index	Peak Type	Area Intg	Area IntgP	Center Grvty	Max Height	FWHM
1	Gaussian	0	0	1750	0.01004	0.04678
2	Gaussian	−4.58955	−12.19146	1736	0.25847	16.6815
3	Gaussian	−9.94357	−26.4136	1723.62622	0.58734	15.9046
4	Gaussian	−4.86841	−12.9322	1711	0.2094	21.8412
5	Gaussian	−4.33661	−11.51954	1699	0.11605	35.1044
6	Gaussian	−13.43769	−35.69521	1688	0.13978	100
7	Gaussian	−0.46981	−1.24799	1655	0.02355	18.77208

Figure S2. unfolded infrared spectrum for S3 (MDI-530PCL-MDI-mPDA) (a) and S8 (MDI-2000PCL-MDI-BAPS) (b). The red line is an approximation using the six peaks of the Gaussian function [44,45].

We obtain that the fraction of hydrogen-bonded urethane groups (C_b) for S3 (MDI-PCL530-MDI-mPDA) is $[3.75 + 1.59]/[1.2 * 5.02 + 3.75 + 1.59] * 100\% = 47.0\%$;

for S8 (MDI-PCL2000-MDI-BAPS) one gets

$$[14.9 + 4.3]/[1.2 * 9.94 + 14.9 + 4.3] * 100\% = 61.7\%.$$

S3. NMR spectroscopy for PUU

To interpret the proton NMR-spectra of the obtained multiblock copolymers, a number of model reactions between the monomers used to form the soft and hard segments were carried out (Figure S3). Copolymers based on 4,4'-MDI and PCL in a ratio of 1:1 were synthesized. The next few model syntheses were carried out in order to describe the signals corresponding to the urea groups. In the first synthesis, 4,4′-MDI and diamine BAPS were used as monomers to obtain polyurea in a 1:1 ratio. The second synthesis simulated a side reaction of the formation of oligomers from 4,4′-MDI molecules when water enters the reaction medium. The third synthesis was carried out to describe the signals of the urea

groups in multiblock copolymers obtained using 2,4-TDI. For this purpose, a reaction was carried out to obtain polyureas based on 2,4-TDI and diamine BAPS at a ratio of 1:1.

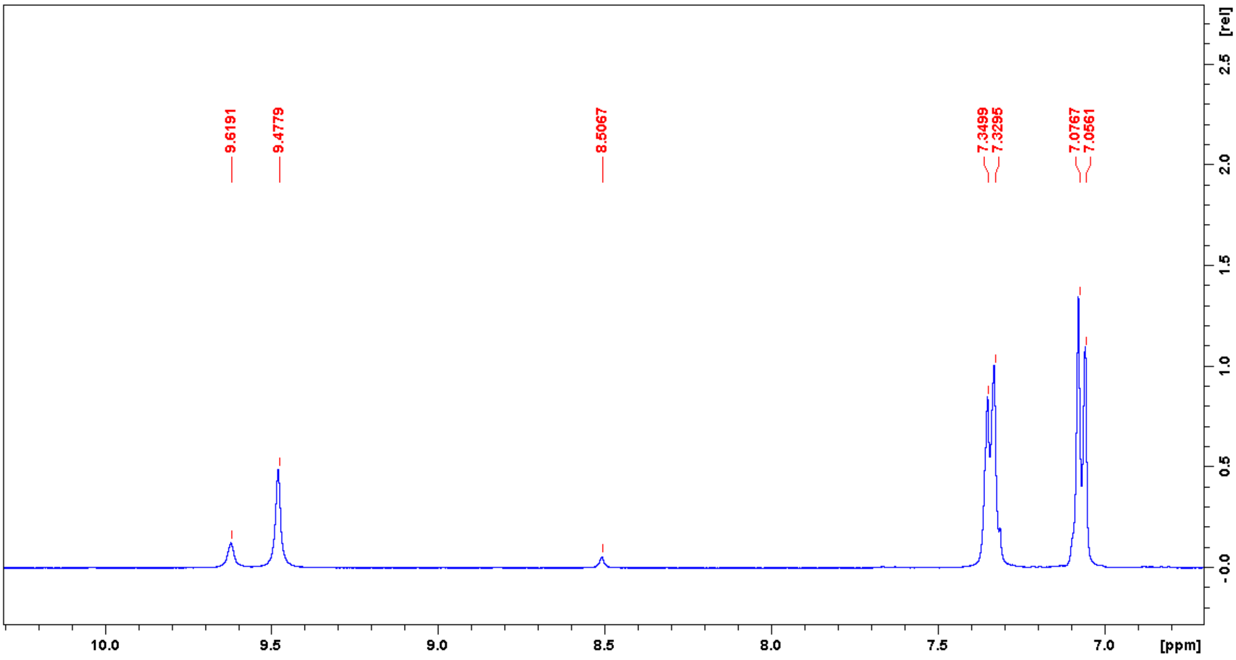

a

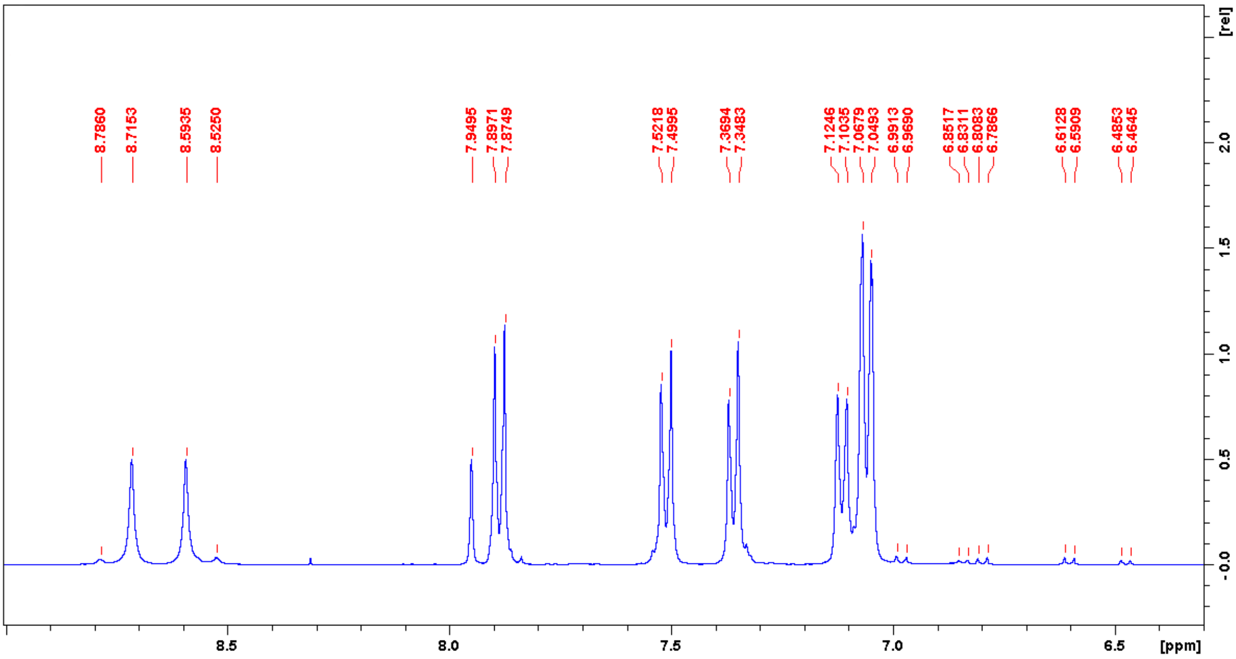

b

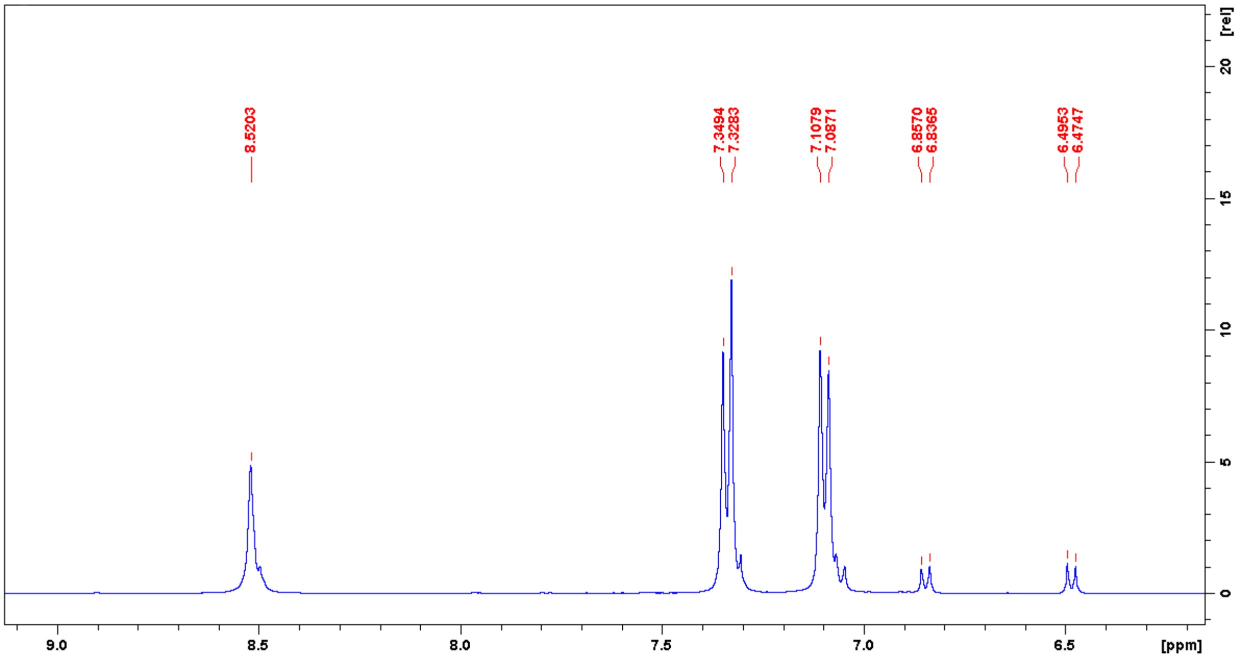

c

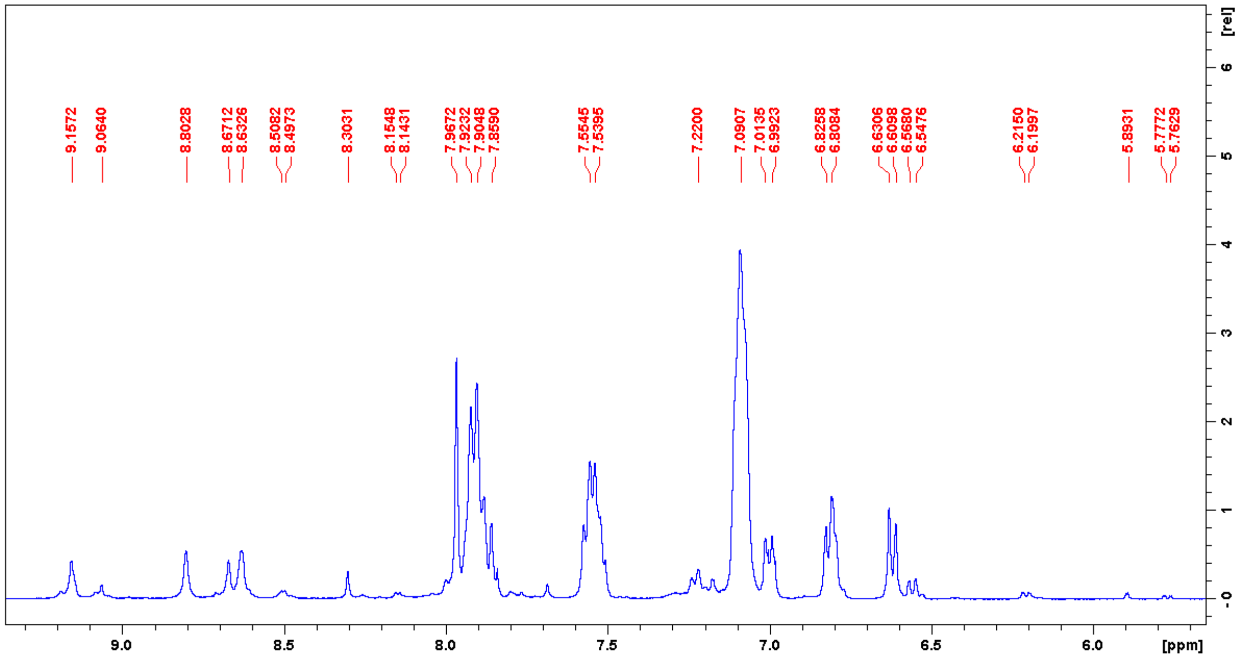

d

Figure S3. ^{1}H NMR spectra of model compounds: 4,4'-MDI-530PCL (a), 4,4'-MDI-BAPS (b), 4,4'-MDI-4,4'-MDI (c), 2,4-TDI-BAPS (d).

S4. Small Angle Scattering Data Analysis

As for the sizes of the hard block concentration regions (hard phase domains), they were estimated using the SANS and SAXS methods. A broad peak model was used to describe the SAS data. This model calculates an empirical functional form for SAS data characterized by a broad scattering peak. Many SAS spectra are characterized by a broad

peak even though they are from amorphous soft materials. For example, soft systems that show a SAS peak include copolymers, polyelectrolytes, multiphase systems, layered structures, etc.

The d-spacing corresponding to the broad peak is a characteristic distance between the scattering inhomogeneities (such as in lamellar, cylindrical, or spherical morphologies, or for bicontinuous structures) [50].

To approximate the SANS data, the model of the broad Lorentzian-type peak at the end of the power-law decay appeared to be of advantage with SasView software [50]. At that, the scattering intensity $I(q)$ is calculated as

$$\mathrm{I}(q) = \frac{A}{q^n} + \frac{C}{1+(|q-q_0|\xi)^m} + \mathrm{B}, \tag{S2}$$

Here the peak position is related to the d-spacing as $q_0 = 2\pi/d_0$. A is the Porod law scale factor, n the Porod exponent, C is the Lorentzian scale factor, m the exponent of q, ξ the screening length, and B the flat background.

Here is the result obtained for the segmented polymeric system S2 (TDI-PCL2000-TDI-mPDA, Table 1 in the main body) (Figure S4).

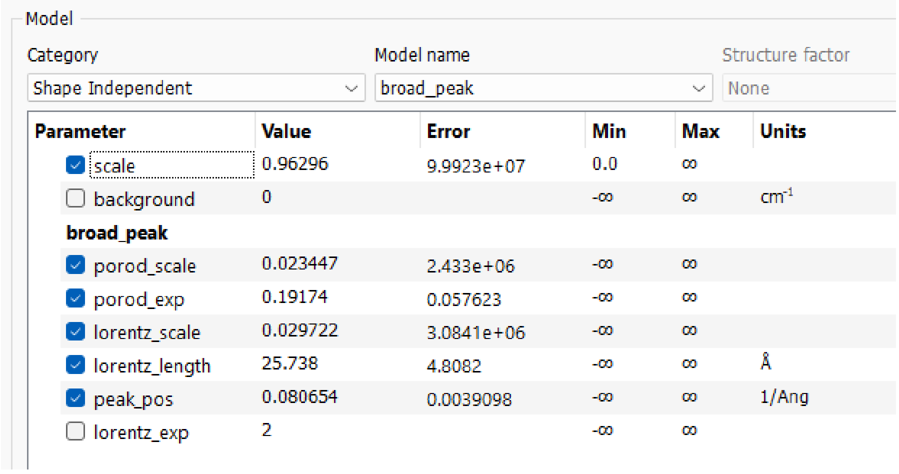
Model

Category: Shape Independent | Model name: broad_peak | Structure factor: None

Parameter	Value	Error	Min	Max	Units
☑ scale	0.96296	9.9923e+07	0.0	∞	
☐ background	0		-∞	∞	cm⁻¹
**broad_peak**					
☑ porod_scale	0.023447	2.433e+06	-∞	∞	
☑ porod_exp	0.19174	0.057623	-∞	∞	
☑ lorentz_scale	0.029722	3.0841e+06	-∞	∞	
☑ lorentz_length	25.738	4.8082	-∞	∞	Å
☑ peak_pos	0.080654	0.0039098	-∞	∞	1/Ang
☐ lorentz_exp	2		-∞	∞	

a

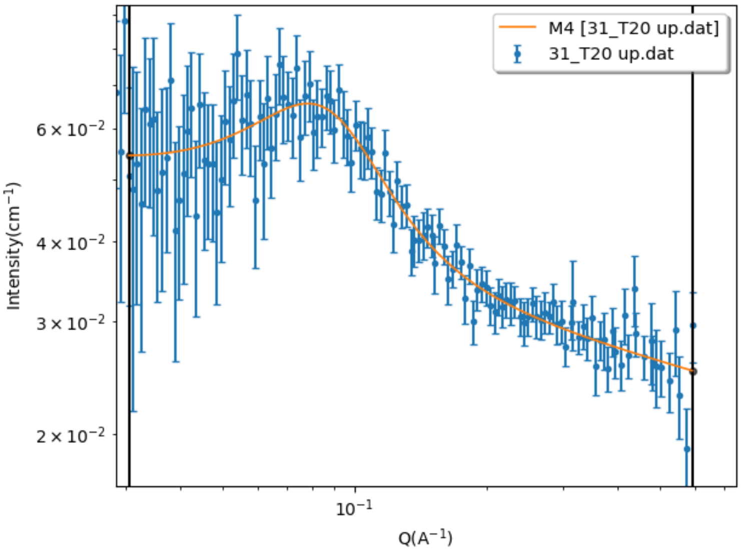

b

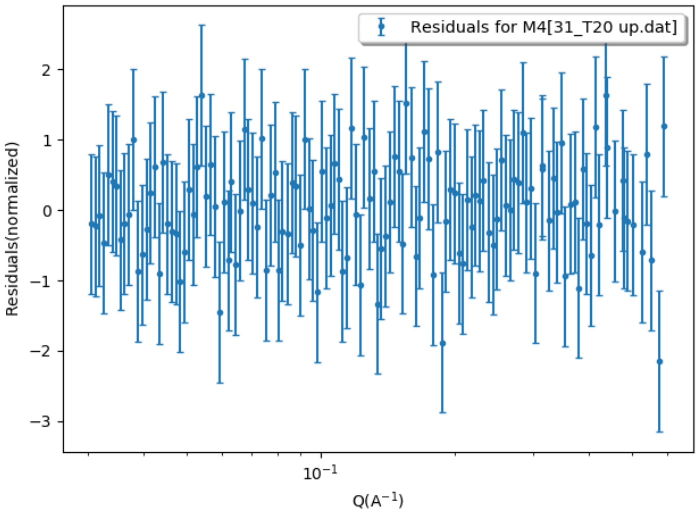

c

Figure S4. Parameters of fitting according the model (Equation (S2)) (**a**). The SANS data for S2 (TDI-2000PCL-TDI-mPDA) (**b**). The orange curve is the fitting upon Equation (S2). The residuals (normalized) for fitting according the model (Equation (S2)) (**c**).

The reduced χ^2 is 0.49897. The distance between the aromatic blocks $d_0 = 2\pi/q_0$ = 6.28/0.080654 = 77.9 Å . The effective radius of the blocks themselves ξ = 25.7 Å . So, their diameter is 51.4 Å .

S5. Shape Memory

The PUU tested in the first thermomechanical cycle show acceptable values (98%) of the shape fixation coefficient (R_f) and low shape recovery coefficients (R_r) (Table S1).

Table S1. Shape memory properties of the segmented PUU

System	Testing in DMA mode	
	R_f	R_r
S2	98.8	54.7
	98.5	69.4
	98.4	86.1
S4	99.5	57
S8	99	25
